# Synergistic Apoptosis-Inducing Effects on A375 Human Melanoma Cells of Natural Borneol and Curcumin

**DOI:** 10.1371/journal.pone.0101277

**Published:** 2014-06-27

**Authors:** Jianping Chen, Lin Li, Jianyu Su, Bing Li, Tianfeng Chen, Yum-Shing Wong

**Affiliations:** 1 College of Light Industry and Food Sciences, South China University of Technology, Guangzhou, China; 2 Department of Chemistry, Jinan University, Guangzhou, China; 3 School of Life Sciences, The Chinese University of Hong Kong, Hong Kong SAR, China; 4 Guangdong Hua Qing Yuan Biological Technology Co., Ltd., Meizhou, China; Sudbury Regional Hospital, Canada

## Abstract

This study was to investigate the synergistic effect of NB/Cur on growth and apoptosis in A375 human melanoma cell line by MTT assay, flow cytometry and Western blotting. Our results demonstrated that NB effectively synergized with Cur to enhance its antiproliferative activity on A375 human melanoma cells by induction of apoptosis, as evidenced by an increase in sub-G1 cell population, DNA fragmentation, PARP cleavage and caspase activation. Further mechanistic studies by Western blotting showed that after treatment of the cells with NB/Cur, up-regulation of the expression level of phosphorylated JNK and down-regulation of the expression level of phosphorylated ERK and Akt contributed to A375 cells apoptosis. Moreover, NB also potentiated Cur to trigger intracellular ROS overproduction and the DNA damage with up-regulation of the expression level of phosphorylated ATM, phosphorylated Brca1 and phosphorylated p53. The results indicate the combinational application potential of NB and Cur in treatments of cancers.

## Introduction

**Figure 1:**
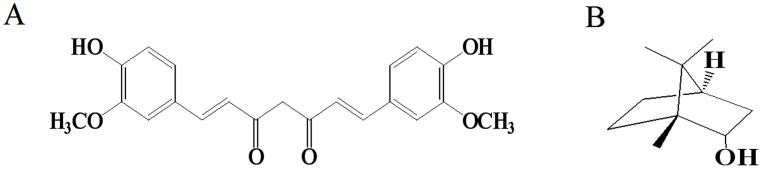
The structure of curcumin (A) and nature borneol (B).

Curcumin (Cur), bis(4-hydroxy-3-methoxy-phenyl)-1,6-heptadiene-3,5-dione, shown in **Figure. 1A**, is an active ingredient from the rhizome of the plant, *Curcuma longa*, which is widely used in food industry as a spice, coloring agent, food preservative and in traditional medicine as an ingredient [1]–[3]. In recent decades, many studies demonstrated that Cur possessed wide-ranging antioxidant, anti-inflammatory, anti-proliferative, anti-angiogenic and anti-cancer activities [4]–[5]. Numerous studies reported the anticancer effect of Cur on a wide variety of cancer cell lines, including hepatocellular carcinoma cell lines [6], thyroid cancer cells [3], colon cancer cells [7]–[9], breast cancer cells [10], bladder cancer cells [11], ovarian cancer cells [5], [12] and so on. In spite of its bioactivities, its application is extremely limited because of its low bioavailability related to its insolubility in water as well as its poor absorption and rapid metabolism [13]. To enhance the bioavailability of Cur, various methods such as preparation of nanoparticles, liposomes, micelles, phospholipid complexs, and structural analogues of Cur were used [14], [15]. Moreover, combination of Cur and other drugs was also reported to improve the bioavailability of Cur due to increasing the cellular uptake of Cur [15]. Therefore, enhancement of the cellular uptake of Cur could be a strategy to improve its anti-cancer activities.

Borneol (C_10_H_18_O), a terpene and bicyclic organic compound, has been widely used in the food and drug industries as an aromatic spice and a valuable medical material. Generally, borneol is classified as synthetic borneol (SB) and natural borneol (NB). SB is composed of (+)-Borneol and (−)-isoborneol, while NB only includes (+)-borneol. Researchers have reported that isoborneol displayed more mucosa stimulus and hepatotoxicity than (+)-borneol [16]. Therefore, as a safer form of borneol, NB, shown in **Figure. 1B**, has the potential for use in commercial applications and current research based on a comprehensive evaluation. Recently, researchers have shown that NB is a very effective penetration enhancer, as evidenced by improving bioavailability of drugs, accelerating the opening of blood-brain barrier (BBB) and enhancing the distribution of drugs in brain tissue [16]–[19]. For example, Cai *et al.* reported that NB increased the absorption of gastrodin in the gastrointestinal tract and the bioavailability of gastrodin in the brain [18]. Moreover, Zhou *et al.* also reported that NB significantly improved the intestinal absorption of akebia saponin D [20]. Some mechanisms such as improvement of cell membrane permeability, loosening of tight junctions and loss of mucus viscosity and elasticity have been proposed to explain this potency [20]–[21]. Therefore, NB would possibly make a good penetration enhancer for the cellular uptake of Cur.

Malignant melanoma is the deadliest form of skin cancer which possesses rapidly spreading and high invasive capacity [22]. It was estimated that there were 47,700 cases of invasive malignant melanoma happened in 2000 year and 20,000 to 40,000 cases of melanoma in-situ newly diagnosed in the US, and 7,700 persons died from malignant melanoma in the US every year, which caused heavy public health burden in the US and worldwide [23]. To date, there is still short of effective systemic therapies for this disease because of the drug resistance in malignant melanoma. Consequently, in this respect, it is necessary to develop new effective drugs which can selectively kill malignant melanoma cells by overcoming drug resistance. Up to now, due to reducing the dose of chemotherapeutic drugs and the side effects, the combination chemotherapy is regarded as a high-efficiency method.

Therefore, in the present study, we aimed to investigate the ability of NB to synergize with Cur to induce A375 human melanoma cell apoptosis, and to elucidate the underlying molecular mechanisms to explain the synergistic effects. This study demonstrated that NB could be the potential to further develop a chemosensitizer of Cur in treatment of human cancers.

## Materials and Methods

### 1. Materials

Curcumin, thiazolyl blue tetrazolium bromide (MTT), propidium iodide (PI), 4′, 6-Diamidino-2-phenyindole (DAPI), dihydroethidium (DHE), and bicinchoninic acid (BCA) kit were purchased from Sigma-Aldrich (Sigma-Aldrich, St Louis, MO) and natural borneol (NB) was obtained from the Natural Institute for the Control of Pharmaceuticals and Biological Products, Beijing, China. Substrate for caspase-3 (Ac-DEVD-AMC), caspase-8 (Ac-IETD-AFC) and caspase-9 (Ac-LEHDAFC) were purchased from Calbiochem (Darmstadt, Germany). The antibodies against caspase-3 (9662), caspase-8 (4790), caspase-9 (9502), PARP (9542), phospho/total-p53 (9286/9282), phosphor/total-ERK (4377S/4695), phospho/total-Akt (4058L/3788S), phospho-JNK (4668P), and β-actin (4970S) were purchased from Cell Signaling Technology (Beverly, MA). The water used in all experiments was ultrapure by a Milli-Q water purification system from Millipore.

### 2. Cell Culture

A375 human melanoma cell line was purchased from American Type Culture Collection (ATCC, Manassas, VA). A375 cells were maintained in DMEM medium supplemented with fetal bovine serum (10%), penicillin (100 units/ml) and streptomycin (50 units/ml) at 37°C in a humidified (5% CO_2_, 95% air) atmosphere.

### 3. Drug treatment, MTT assay and cellular uptake

A375 cells with a density of 1.5×10^4^ cells/well were seeded in 96-well culture plates for 24 h. For the concentration effect, the cells were pre-treated with NB (40 µg/ml) for 12 h and co-incubated with different concentrations of Cur for another 72 h to examine the synergistic effects of NB/Cur on A375 cells. As a negative control, the cells were only treated with different concentrations of Cur for another 72 h. Cell viability was determined by MTT assay as previously described [24]. ie, after treatment of the cells, 20 µl/well MTT solution (5 mg/ml in PBS) was added and incubated for 4 h. Then the medium was aspirated and replaced with 150 µl/well dimethylsulfoxide to dissolve the formazan salt. The color intensity of the formazan solution, which reflects the cell growth condition, was measured at 570 nm using a microplate spectrophotometer (Versamax). The cell viability was expressed as a percentage of control.

For quantitative analysis of cellular uptake, the A375 cells with a density of 8×10^4^ cells/well were seeded in 96-well culture plates and incubated at 37°C in CO_2_ incubator. After 24 h, the cells were exposed to 40 µg/ml NB, 20 µM Cur, and NB (40 µg/ml)/Cur (20 µM), respectively and then incubated for various periods of time. Control used was untreated cells. At the end of the incubation, the medium was removed from the wells and the cells were rinsed three times with cold PBS (Gibco) to remove the NB or Cur outside the cells. After that, 200 µL of 1% Triton X-100 (Sigma-Aldrich) in 0.1 M NaOH (Sigma-Aldrich) solution was added to lyse the cells. The concentrations of intracellular Cur were determined by using a fluorescence microplate reader (Spectra Max M5, Sunnyvale, California) with excitation and emission wavelengths set at 425 and 545 nm, respectively.

### 4. Flow Cytometric Analysis

The cell cycle distribution was monitored by flow cytometric analysis as previously described by Chen et al. [24]. ie, A375 cells were harvested after treatments with NB alone, Cur alone, or NB/Cur for 72 h, washed with PBS and fixed with 75% ethanol at −20°C overnight. The fixed cells were then stained with 500 µl propidium iodide (PI) working solutions (1.21 mg/mL Tris, 700 U/ml RNase, 50.1 mg/ml propidium iodide, pH 8.0) for 4 h in darkness. The stained cells were performed by an Epics XL-MCL flow cytometer (Beckman Coulter, Miami, FL) and the cell cycle distribution was analyzed using MultiCycle software (Phoenix Flow Systems, San Diego, CA). The proportion of cells in G0/G1, S, G2/M phases was represented as DNA histograms. Apoptotic cells with hypodiploid DNA content were measured by quantifying the sub-G1 peak in the cell cycle pattern. For each experiment 10000 events per sample were recorded.

### 5. TUNEL and DAPI Staining

Tunnel and DAPI Staining assay was carried out according to the method described by Chen et al. [24]. ie, Cells cultured in chamber slides were fixed with 37% formaldehyde for 10 min and then permeabilized with 0.1% Triton X-100 in PBS. After that, the cells were incubated with a 100 µl/well TUNEL reaction mixture containing nucleotide mixture and terminal deoxynucleotidyl transferase (TdT) for 1 h and then with 1 µg/mL of DAPI for 15 min at 37°C for nuclear staining. The Stained cells were washed with PBS and examined on a fluorescence microscope (Nikon Eclipse 80i).

### 6. Caspase Activity Assay

In brief, the harvested cell pellets were suspended in RIPA cell lysis buffer (50 mM Tris-HCl (pH 7.4), 150 mM NaCl, 1% Nonidet P-40, and 0.1% SDS) and incubated on ice for 1 h. After centrifugation at 11000 g for 30 min, supernatants were collected and immediately measured for protein concentration by a BCA kit. The cell lysates were placed in 96-well plates and then the specific caspase substrates (Ac-DEVD-AM C for caspase-3, Ac-IETD-AMC for caspase-8, and Ac-LEHD-AM C for caspase-9) were added. Plates were then incubated at 37°C for 1 h, and caspase activity was determined by the fluorescence intensity with the excitation and emission wavelengths set at 380 and 440 nm, respectively.

### 7. Measurement of ROS generation

The relative level of intracellular ROS generation was analyzed by DHE fluorescence assay. ie, the cells were harvested by centrifugation, washed twice with PBS, and suspended in PBS (1×10^6^ cells/ml) containing 10 µM of DHE. After incubation at 37°C for 30 min, the cells were collected and resuspended in 100 µl PBS. Then A375 cells were seeded in 96-well microplates at 100×10^4^ cells/well, and the cells were then separately incubated with 40 µg/ml NB, 20 µM Cur, and combination of 40 µg/ml NB and 20 µM Cur at 37°C for 5, 10, 15, 30, 60, 90, 120 min, respectively. The ROS level was then immediately measured by a Tecan Safire multifunctional mono-chromator based microplate reader (Tecan, Switzerland). The generation of ROS was determined by fluorescence intensity with the excitation and emission wavelengths set at 300 and 610 nm respectively. Relative DHE fluorescence intensity of treated cells was expressed as percentage of control (as 100%).

### 8. Western Blot Analysis

Total cellular proteins were harvested by incubating the cells in the RIPA lysis buffer (50 mM Tris-HCl (pH 7.4), 150 mM NaCl, 1% Nonidet P-40, and 0.1% SDS) obtained from Cell Signaling Technology. The protein concentrations were determined by BCA kit (Sigma-Aldrich) according to the manufacturer's protocols. SDS-PAGE was performed in 10% tricine gels with equal amounts of protein loaded per lane. After electrophoresis, proteins were transferred from the gel to a nitrocellulose membrane at 110 V for 1 hour, and then the membrane was blocked with 5% nonfat milk in TBST buffer (20 mM Tris-HCl, pH 7.4, 137 mM NaCl, and 0.1% Tween-20) for 1 hour. The membranes were then incubated with primary antibodies at 1∶1000 dilution in 5% nonfat milk over night at 4°C, followed by secondary antibodies conjugated with horseradish peroxidase at 1∶2000 dilution for 1 hour at room temperature. Protein bands were visualized on X-ray film using an enhanced chemiluminescence system (Kodak).

### 9. Statistical Analysis

Experiments were carried out at least in triplicate and repeated three times. All data were expressed as mean ±SD. Statistical analysis was performed using SPSS statistical package (SPSS 130 for Windows, SPSS, Inc Chicago, IL). The difference between two groups was analyzed by two-tailed Student's t-test. Differences with *P*<0.05 (*) or *P*<0.01 (**) was considered statistically significant. Bars with different characters are statistically different at *P*<0.05 level.

## Results and Discussion

### 1. Synergistic effects of NB/Cur on A375 human melanoma cells viability

**Figure 2:**
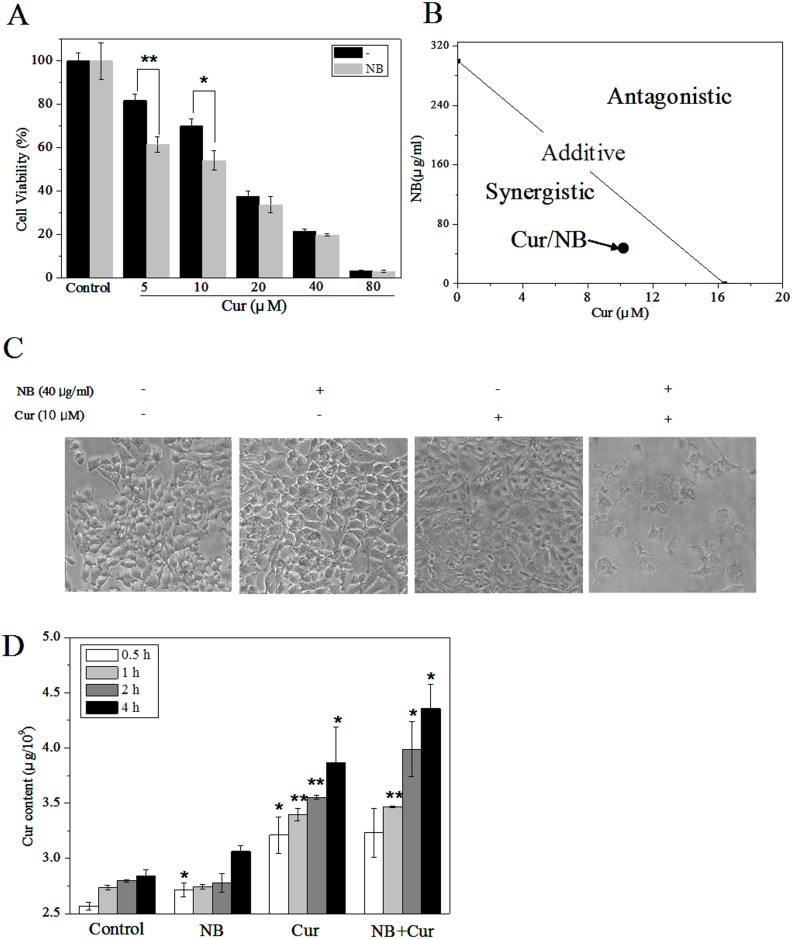
The synergistic effects of NB/Cur on A375 cells. (A) A375 Cells were pretreated with or without 40 µg/ml NB for 12 h, then treated with the different concentration of Cur and incubated for 72 h. Cell viability was examined by MTT assay. Bars between cell pretreated with NB and without NB are significantly different at *P*<0.05(*) or *P*<0.01(**) level. (B) Isobologram analysis of the antiproliferative effects of NB and Cur on A375 cells. (C) Morphological image of cells after pretreatment of NB for 12 h then incubated with Cur for 72 h as examined by phase-contrast microscopy (magnification, 200×). All images shown here are representative of three independent experiments with similar results. (D) Cellular uptake of Cur. Quantitative analysis of Cur concentrations in A375 cells exposed to NB (40 µg/ml) and Cur (20 µM) alone and in their combination for 0.5, 1.0, 2.0, 4.0 h, respectively. Bars between treatment groups and control groups are significantly different at *P*<0.05(*) or *P*<0.01(**) level.

Firstly, we used the MTT assay to investigate the ability of NB to synergize with Cur to induce A375 cancer cell apoptosis. As shown in **Figure. 2A**, Cur alone decreased the cell viability in a dose-dependent manner. For example, treatment with 10 µM Cur for 72 h decreased the cell viability to 70% in comparision to control cells. However, this cytotoxic effect was significantly strengthened by pretreatment with NB. At concentration of 40 µg/ml, NB reduced the cell viability of 10 µM Cur-treated cells from 70% to 54.20%. These results indicated that pretreatment of the cells with NB (40 µg/ml) caused significantly stronger cell growth inhibition than Cur alone, as evidenced by a decrease in IC_50_ of Cur from 16.4 to 9.5 µM. Moreover, pretreatment of the cells with 40 µg/ml NB for 12 h followed by simultaneous incubation with 10 µM Cur for 72 h resulted in much stronger growth inhibition by comparing with Cur and NB alone. This result was also confirmed by microscopic examination (**Figure. 2C**). The images showed cells treated with NB and Cur occurred more cell shrinkage, cell rounding, and formation of apoptotic bodies than cells treated with Cur alone for 72 h. In contrast, cells treated with NB alone for 72 h and control cells treated without NB and Cur remained intact with regularity in shape.

To examine whether the anti-tumour action of NB and Cur in A375 human melanoma cells were synergistic, addictive, or antagonistic, the growth inhibitory effects were analyzed by the isobologram method [25]. As shown in **Figure. 2B**, the IC_50_ values of NB and Cur on A375 cells were found at 300 µg/ml and 16.40 µM, respectively, whereas the IC_50_ value for the combined treatments of NB and Cur was 9.5 µM. The results of the isobologram analysis revealed that the growth inhibitory effects between NB and Cur was strongly synergistic, as evidenced by the location of the data point in the isobologram being far below the line defining an additive effect. Moreover, the actual IC_50_ value of the combination (9.5 µM) was significantly lower than the theoretical ones (14.21 µM). The combination index (CI) of the IC_50_ value of the NB and Cur was found at 0.67, which further confirmed the synergism between NB and Cur.

To evaluate the effects of NB on Cur uptake, the cellular uptake of Cur was measured by employing fluorescent spectrometry. As shown in **Figure. 2D**, comparing with the control group, treatment of A375 cells with NB (40 µg/ml) for 4 h showed no obvious effects on the intracellular Cur concentration. However, the combination treatment of Cur and NB (concentration calculated by Cur) significantly increased the Cur concentration in a time dependent manner. For instance, after 0.5, 1.0, 2.0 and 4.0 h incubation with NB and Cur, the intracellular concentrations of Cur increased from 2.57 (control) up to 3.23, 3.47, 3.99 and 4.36 µg/10^9^ cells, which was about 1.01, 1.02, 1.12, 1.13 times higher than the group of Cur without NB (3.21, 3.40, 3.55, 3.86 µg/10^9^ cells). These results demonstrated NB enhanced the cellular uptake of Cur in A375 cells. Taken together, our results clearly demonstrated that the strategy to combine NB and Cur could be a highly efficient way to enhance anticancer activities of Cur.

### 2. NB synergizes Cur to induce A375 apoptosis with the involvement of DNA damage

**Figure 3:**
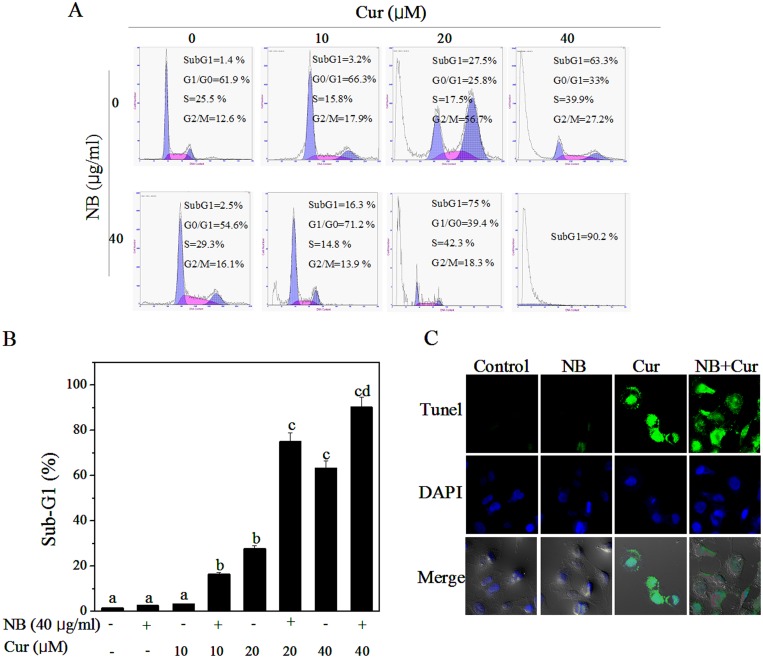
The Synergistic induction effects of apoptotic A375 cell death by NB/Cur. (A) Fow cytometric analysis of A375 cells treated with NB alone, Cur alone and NB/Cur for 72 h, respectively. Hypodiploid DNA content of apoptotic cells was measured by quantifying the Sub-G1 peak. (B) Quantitative analysis of NB/Cur induced apoptotic A375 cell death by measuring the sub-G1 cell population. Bars with different characters are statistically different at *P*<0.05 level. (C) Representative photomicrographs of DNA fragmentation and nuclear condensation in response to NB and/or Cur treatment, as measured by TUNEL assay and DAPI staining. A375 Cells were exposed to 40 µg/ml NB for 12 hours and then treated with 20 µM Cur for another 24 hours. Top images group shows the results which DNA fragmentation was examined by TUNEL assay (magnification, 200×). TUNEL-positive nuclei because of DNA fragmentation emerges as green color. Middle images group shows the results which apoptosis in A375 cells was detected by DAPI staining (magnification, 200×). Bottom images groups shows the results which images of DAPI staining and TUNEL for the same area were merged (Magnification, 200×). All data here are expressed as means ± SD of triplicates. All images shown here are representative of three independent experiments with similar results.

Inhibition of proliferation in cancer cells could be the result of induction of apoptosis or cell cycle arrest or a combination of these two modes. In order to confirm the mechanisms of action of NB/ Cur that caused cell death, we carried out a PI-flow cytometric analysis to examine the apoptotic Sub-G1 fraction in the treated cells. As shown in **Figure. 3A and 3B**, no significant apoptosis was observed in cells exposed to NB (40 µg/ml) alone. Treatment of Cur alone increased the percentage of apoptotic cells from 1.4% (control) to 3.2% (10 µM), 27.5% (20 µM) and 63.3% (40 µM), respectively. However, comparing with the treatment of Cur alone, the combined treatment with Cur (10, 20, 40 µM) and NB (40 µg/ml) significantly increased the cell apoptosis from 3.2% to 16.3%, 27.5% to 75%, and 63.3% to 90.2%, respectively. To further confirm the induction of apoptosis, we detected the DNA fragmentation and nuclear condensation as apoptotic markers by TUNEL and DAPI co-staining assay. As shown in **Figure. 3C**, the cells treated with NB and Cur in combination showed significant DNA fragmentation and nuclear condensation, which were not detected in the cells treated with NB or Cur alone. These results indicated that apoptosis was the major mode of cell death induced by combined treatment of NB and Cur.

### 3. NB synergizes Cur to induce A375 apoptosis by activating caspase family protein

**Figure 4:**
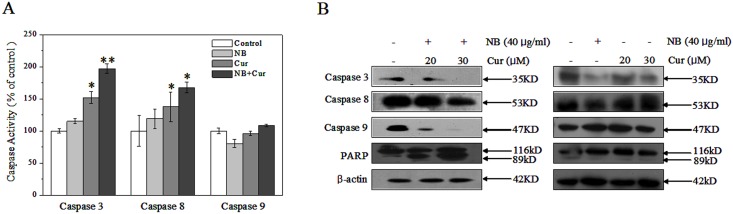
Effects of NB/Cur on caspase family proteins and PARP proteins in A375 cells. (A) Caspase activities as measured by specific fluorescent substrates for caspase-3/8/9. Cells were pretreated with 40 µg/ml NB for 12 h and then treated with 20 µM Cur for 72 h, respectively. Significant difference between treatment groups and control groups is indicated at *P*<0.05 (_*_) or *P*<0.01 (_**_) level. (B) Western blot analysis of caspases activation and PARP cleavage in A375 cells pretreated with or without 40 µg/ml NB for 24 h and then treated with or without 20 µM and 30 µM Cur for 72 h, respectively. All results shown here are representative of three independent experiments with similar results.

Apoptosis, a morphologically distinct form of cell death resulting from the activation of cell suicide program, plays a very important role to maintain the integrity and homeostasis of multicellular organisms [14], [26]–[27]. Two most important pathways involved in cells apoptosis are the death receptor-mediated (extrinsic) pathway and the mitochondrial-mediated (intrinsic) pathway. Caspases, a family of cysteine acid proteases, play a pivotal role in two central apoptotic mechanisms [14]. Caspase-3 is the final phase of apoptosis execution shared by both pathways. Caspase-8 is a major initiator caspase in this extrinsic apoptotic pathway mediated by the death receptors. In contrast, Caspase-9 is a predominant initiator in the intrinsic pathway mediated by mitochondria. Many studies have showed that Cur induced injury to human ovarian cancer cells through increasing activation of caspase-3 and cleavage of PARP [14]. In this present study, to determine whether caspases-3/8/9 and their substrates, poly (ADP-ribose) polymerase (PARP), were also involved in this process, we firstly investigated the activation of caspase-3/8/9. In agreement with the previous studies, our results showed the treatments of NB (40 µg/mL) alone or Cur (20 µM) alone decreased the activation of caspase-9 and slightly increased the activation of caspase-3/8. However, comparing with each drug alone, the combined treatment of NB (40 µg/mL) and Cur (20 µM) slightly increased the activation of caspase-9 and significantly increased the activation of caspase-3/8 in a dose-dependant manner, indicating that both extrinsic death receptor-mediated and intrinsic mitochondria-mediated apoptotic pathways were involved in cell apoptosis (**Figure. 4A**). Meanwhile, these results were further confirmed by cleavage of caspases and PARP as examined by Western blotting. As shown in **Figure. 4B**, exposure of A375 cells to combined treatment of NB and Cur resulted in cleavage of caspase-3/8/9, which subsequently induced the proteolytic cleavage of PARP, a protein serving as a biochemical hallmark of cells undergoing apoptosis. However, Cur or NB alone showed only a slight decrease in the activation of caspase-3 and no effects on caspase-8/9 and PARP activation. Taken together, these results demonstrated that both the extrinsic and intrinsic apoptosis pathways were involved in the combined treatment-induced apoptosis in A375 cells.

### 4. NB synergizes Cur to induce A375 apoptosis by DNA damage and with the involvement of Akt and MAPKs pathways

**Figure 5:**
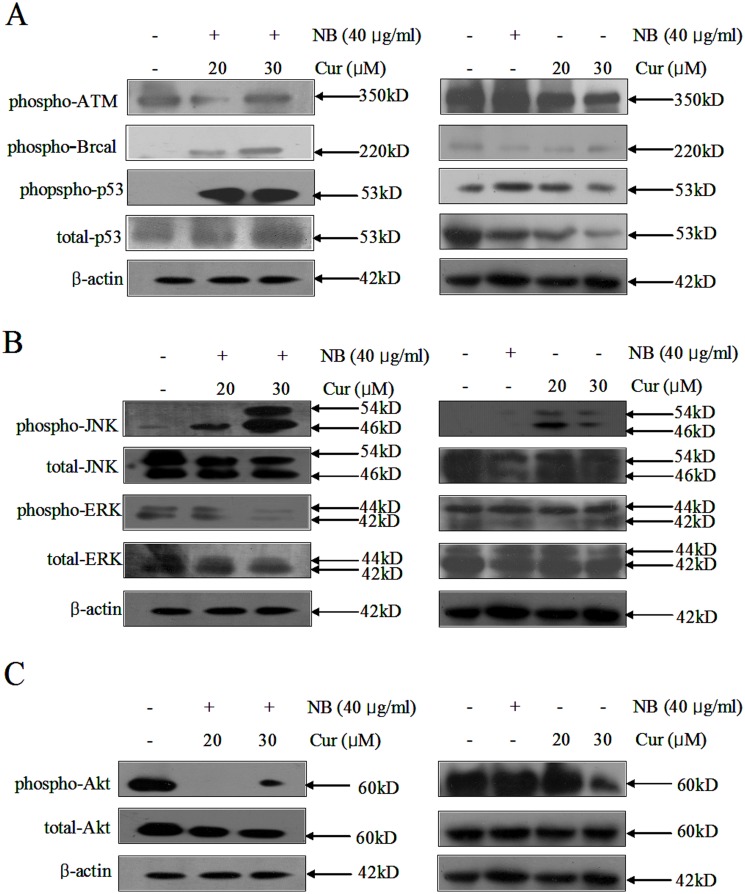
Effects of NB/Cur on induction of DNA damage, MAPK and Akt signaling pathways in A375 cells. Cells were pretreated with or without 40 µg/ml NB for 12 h and incubated with or without 20 µM and 30 µM Cur for 72 h, respectively. (A) The phosphorylation status and expression levels of ATM, Brca1 and p53 were detected by Western blotting method. (B) The expression levels of the phosphorylated and total JNK and ERK was examined by Western blotting assay. (C) The expression level of phosphorylated AKt and total Akt were measured by Western blotting assay.

Studies demonstrated that DNA damage could trigger several signal transduction pathways that led either to damage repair and reduction of cell cycle progression or to apoptosis [28]. Meanwhile, DNA damage triggers cells apoptosis mainly by activation of the extrinsic death receptor apoptosis pathway and/or the intrinsic mitochondrial apoptosis pathway [29], [30]. ATM (ataxia telangiectasia mutated), ATR (ataxia telangiectasia mutated and rad3-related) and DNA-PK (DNA-dependent protein kinase) are regards as the main ‘players’ of DNA damage recognition and phosphorylated ATM, ATR and DNA-PK can induce the DNA damage response (DDR) [30]. ATM (ataxia telangiectasia mutated) is the core of the DNA damage signaling apparatus and is activated by DNA damage [31]. Meanwhile, p53 and Brca1 (breast cancer gene 1) are also phosphorylated in response to DNA damage, and are the phosphorylate of ATM substrates [32]. Moreover, Shieh et al. also reported that DNA damage can induce phosphorylation of p53 at Serine 15 [33]. Therefore, the expression levels of total and phosphorylated p53 at Ser15 site, phosphorylated ATM and phosphorylated Brca1 were measured by immunoblotting assay. Our results revealed that NB and Cur in combination markedly up-regulated the expression level of total and phosphorylated p53, phosphorylated ATM, and phosphorylated Brca1 in A375 cells in a dose-dependent manner (**Figure. 5A**), which could not be observed in A375 cells expose to Cur or NB alone. These results indicated that the enhancement of DNA damage which contributes to the synergistic effects of NB and Cur on A375 cells apoptosis.

Meanwhile, studies also have shown that the MAPK (mitogen-activated protein kinases) signaling pathway plays a critical role in the action of chemotherapeutic drugs [34] and regulating cell cycle progression and death or growth. JNK (Jun amino terminal kinases), ERK (extracellular regulated protein kinases) and p38MAPK pathway is the three most important pathways in MAPK pathway. JNK and p38MAPK, stress-activated MAPKs, promoted apoptosis, whereas ERK, mitogen-activated MAPKs, could prevent cell apoptosis by blocking the activation of caspases [35]. Moreover, studies have shown that DNA damage also activate other cellular response pathways, such as the (JNK) pathway [36]. Therefore, we next determined whether the MAPK pathway were activated in NB/Cur-treated A375 cells by Western blot analysis using specific antibodies against the phosphorylated (activated) forms of the kinases. Our resulted showed that NB exhibited no effect on the expression of phosphorylation of JNK and ERK, while slight elevation of phosphorylation of JNK and phosphorylation of ERK1/2 were observed in cells exposed to Cur alone. However, exposure of A375 cell to NB/Cur significantly enhanced the expression level of phosphorylation of JNK and decreased the expression level of phosphorylation of ERK1/2 in a dose-dependent manner, indicating that phosphorylation of JNK played a key role in promoting apoptosis (**Figure. 5B**).

The Akt signaling pathway also played a pivotal role in cell proliferation and cell apoptosis by transferring the signals to prevent apoptosis [37], [38]. Huang et al. reported that the abnormalities of Akt signaling in ovarian cancer may be used to predict patient outcomes [39]. Manning et al. reported that Akt could improve the viabilities of cells by arresting the expression of pro-apoptotic proteins, such as BAD, FOXO, p53, GSK3 isoforms and caspase-9 [40]. Therefore, regulation of the Akt signaling pathway may be used as a promising method for curing cancers. In this present study, our results showed NB alone exhibited no obvious change on the expression of phosphorylation of Akt, while the expression levels of phosphorylation of Akt was slightly decreased in cells exposed to Cur alone. However, exposure of A375 cells to NB/Cur significantly decreased the expression levels of phosphorylated Akt and total Akt (**Figure. 5C**), indicating that Cur induces A375 cancer cells death through inhibition of the Akt pathway. Taken together, NB/Cur induce A375 apoptosis with involvement of down-regulation of Akt and ERK1/2 phosphorylation and up-regulation of phosphorylated JNK.

### 5. NB synergizes Cur to induce cell apoptosis via ROS generation

**Figure 6:**
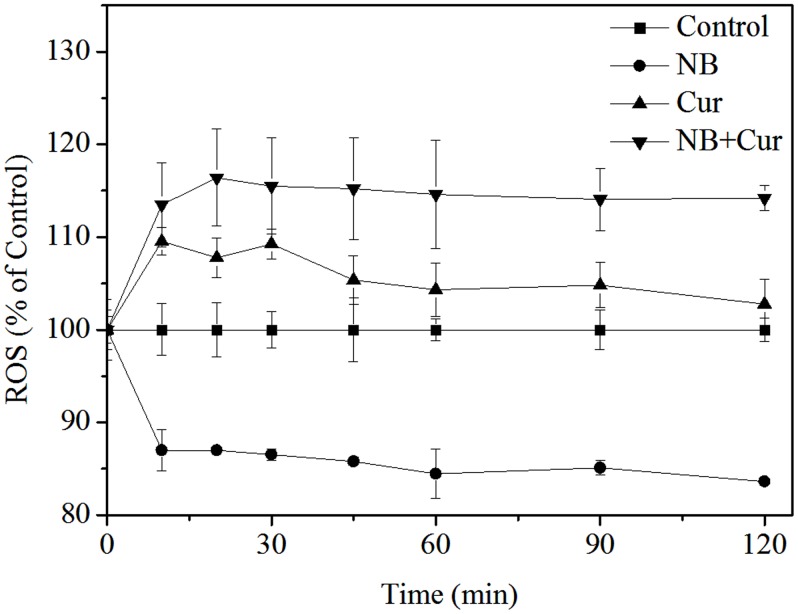
Effects of NB/Cur on ROS generation in A375 cells. Cells were treated with 40 µg/ml NB and/or 20 µM Cur at 37°C for different times after treatment with 10 µM DHE for 30 min and the levels of the intracellular ROS were analyzed by measuring the fluorescence intensity of an oxidation-sensitive fluorescein DHE.

Currently, ROS (reactive oxygen species), such as hydrogen peroxide, superoxide, and hydroxyl radicals, has been regarded as important regulator of apoptosis by regulating signaling pathways including the extrinsic and intrinsic apoptosis pathways and overproduction of ROS in the cells often results in the accumulation of DNA damage [41]–[43]. Martindale et al. also reported that ROS induced the activation of multiple signaling pathways containing Akt and ERK pathway, which resulted in cell survival or death [44]. And it has been reported that ROS triggers down-regulation of the ERK and Akt pathway [45]–[46]. Therefore, we decided to detect whether NB can synergize with Cur to trigger ROS generation by measuring the DHE fluorescence intensity. As is shown in **Figure. 6**, A375 cells treated with NB alone decreased the ROS level. Moreover, a slight enhancement of ROS occurred in the A375 cells treated with Cur. However, cells exposed to NB/Cur significantly increased in intracellular ROS generation in a time-dependent manner, suggesting that ROS may be upstream of apoptotic signaling pathway.

**Figure 7:**
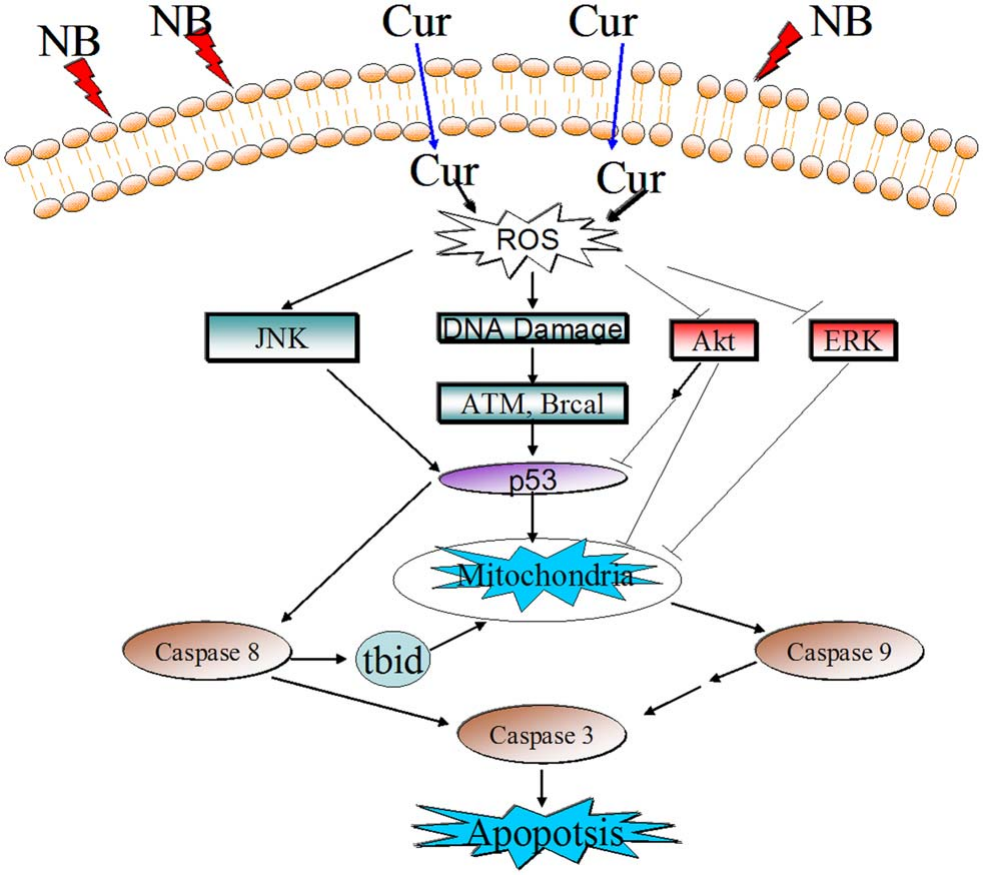
The proposed signaling pathway triggered by NB and Cur in combination in A375 cells. NB increases the cellular uptake of Cur, which subsequently improves intracellular ROS generation. Overproduction of ROS leads to accumulation of DNA damage. DNA damage activates p53 pathway, which causes mitochondrial dysfunction. Mitochondrial dysfunction activates several caspase cascades, and finally leads to cell apoptosis. In addition, ROS also enhances the phosphorylation of JNK and inactivation of Akt and ERK, which enhances the p53 activation and promotes the apoptotic cascade.

**Figure 8:**
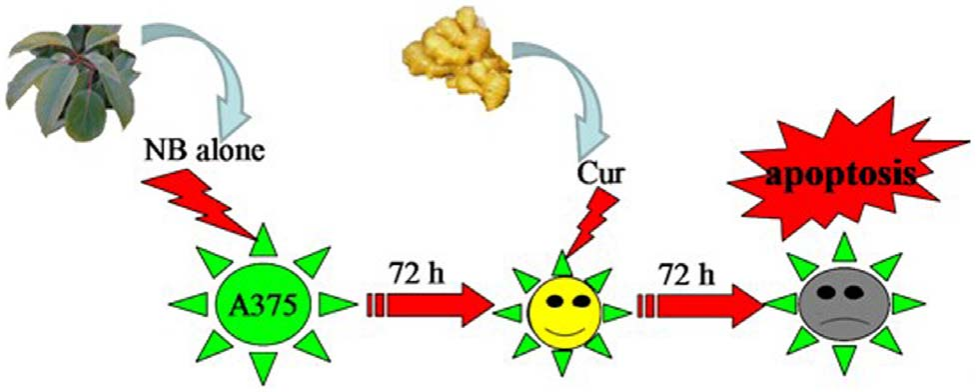
Schematic diagram of the effects of NB/Cur on A375 cells. After alone NB treated A375 cells for 72

Based on the above results, we proposed a signaling network for the synergistic action of NB and Cur. As depicted in **Figure 7**, NB pretreatment enhances intracellular ROS generation. Then, ROS acted as an upstream mediator for JNK, AKT and ERK dephosphorylation, and triggered cell DNA damage, which led to activation of p53 pathway. The activation of p53 pathway causes mitochondrial dysfunction by regulating the expression levels of pro-survival and pro-apoptotic Bcl-2 family members. Mitochondrial dysfunction results in release of apoptogenic factors into cytosol, and then activates caspase 3/8/9, and finally leads to cell apoptosis. In addition, as a functional loop, ROS overproduction lead to inactivation of Akt and apoptosis may be induced through blocking the activation of Akt. In summary, the combination of Cur and NB synergistically inhibited the growth of A375 cells through induction of apoptosis (**Figure 8**).

## Conclusion

Although Cur has no adverse side effect been found in clinical trails [47], the disadvantages such as its low bioavailability have limited its application. Therefore, the development of sensitizers could be a vital strategy to improve the bioavailability of Cur via the enhancement of cellular uptake of Cur in the further. In this present study, we found that NB strongly increased Cur-induced apoptosis in A375 human melanoma cells, as proved by activation of ROS-mediated DNA damage and up-regulation the expression level of phosphorylated JNK and down-regulation the expression level of phosphorylated ERK and Akt. This study suggests that NB could be further developed as a chemosensitizer of Cur in treatment of human cancers and the combination of Cur and NB could be a novel strategy to achieve anticancer synergy.
